# Efficiency of the Adjusted Binary Classification (ABC) Approach in Osteometric Sex Estimation: A Comparative Study of Different Linear Machine Learning Algorithms and Training Sample Sizes

**DOI:** 10.3390/biology11060917

**Published:** 2022-06-15

**Authors:** MennattAllah Hassan Attia, Marwa A. Kholief, Nancy M. Zaghloul, Ivana Kružić, Šimun Anđelinović, Željana Bašić, Ivan Jerković

**Affiliations:** 1 Forensic Medicine and Clinical Toxicology, Faculty of Medicine, Alexandria University, Alexandria 21568, Egypt; marwa.kholief@alexmed.edu.eg; 2 Forensic Medicine and Clinical Toxicology Department, Faculty of Medicine, Misr University for Science and Technology, Giza 3236101, Egypt; drnancyzag@gmail.com; 3 University Department of Forensic Sciences, University of Split, Ruđera Boškovića 33, 21000 Split, Croatia; ivana.kruzic@unist.hr (I.K.); zeljana.basic@unist.hr (Ž.B.); ivanjerkovic13@gmail.com (I.J.); 4 School of Medicine, University of Split, Šoltanska 2, 21000 Split, Croatia; simun.andjelinovic@unist.hr; 5 Clinical Department for Pathology, Legal Medicine and Cytology, University Hospital Center Split, Spinčićeva 1, 21000 Split, Croatia

**Keywords:** machine learning algorithms, adjusted binary classification, osteometric sex estimation, optimal training sample size

## Abstract

**Simple Summary:**

This study adopts a dynamic methodology to explore challenges to the practical application of the adjusted binary classification (ABC) approach, which are related to the unmodifiable characteristics of data used in its development, such as intrasexual variation (sexual dimorphism) of variables and methodological factors such as the selected classification algorithm and sample size. The adequacy of a training dataset’s size was judged relative to the classification performance in an independent test set. Finding an optimal classifier was also addressed in this study, wherein the results demonstrate that both statistical modeling and machine learning techniques perform almost equally in the univariate models; however, differences are evident in the multivariate model due to the different number of variables included via the feature selection process, as well as the effect of inadequate training sample size relative to the test set. This approach is particularly useful when quick classification/prediction is required for making real-time forensic decisions.

**Abstract:**

The adjusted binary classification (ABC) approach was proposed to assure that the binary classification model reaches a particular accuracy level. The present study evaluated the ABC for osteometric sex classification using multiple machine learning (ML) techniques: linear discriminant analysis (LDA), boosted generalized linear model (GLMB), support vector machine (SVM), and logistic regression (LR). We used 13 femoral measurements of 300 individuals from a modern Turkish population sample and split data into two sets: training (*n* = 240) and testing (*n* = 60). Then, the five best-performing measurements were selected for training univariate models, while pools of these variables were used for the multivariable models. ML classifier type did not affect the performance of unadjusted models. The accuracy of univariate models was 82–87%, while that of multivariate models was 89–90%. After applying ABC to the crossvalidation set, the accuracy and the positive and negative predictive values for uni- and multivariate models were ≥95%. Sex could be estimated for 28–75% of individuals using univariate models but with an obvious sexing bias, likely caused by different degrees of sexual dimorphism and between-group overlap. However, using multivariate models, we minimized the bias and properly classified 81–87% of individuals. A similar performance was also noted in the testing sample (except for FEB), with accuracies of 96–100%, and a proportion of classified individuals between 30% and 82% in univariate models, and between 90% and 91% in multivariate models. When considering different training sample sizes, we demonstrated that LR was the most sensitive with limited sample sizes (*n* < 150), while GLMB was the most stable classifier.

## 1. Introduction

The reconstruction of a skeletal biological profile of unidentified human remains encompasses four main parameters: sex, age at death, stature, and population affinity [1,2]. Usually, one of the first steps in that process is osteological identification of sex, as it remarkably reduces the number of possible matches and enables the application of other biological profiling methods that are sex-specific [3]. Sex-related markers in the human skeleton broadly reflect size sexual dimorphism (SSD), general robusticity of bones, and entheseal changes due to muscular bulk attachment and activity [4], but they can also be prominent in shape differences that mainly result from biological adaptations [2,4]. Therefore, two methodological approaches are commonly used for sex diagnosis: morphoscopic and osteometric approaches [2]. The osteometric and morphoscopic features can be successfully analyzed on pelvic and cranial bones [5,6,7,8,9], whereas osteometric methods are commonly most productive using long bones [10,11,12,13,14,15,16,17]. One or multiple skeletal elements or combinations of features enable the forensic deduction of sex depending on the degree of fragmentation and presence of a skeletal element [4,16,17,18,19,20,21]. Undamaged pelvic bones are by far the most accurate bones available for sexing [5]. When the pelvic bones are missing, long bones are considered to have the most important role in sex estimation [22]. Femora were the focus of attention in a multitude of studies owing to their articulation with the pelvis, higher probability of preservation, and function in relation to bipedal motion [10,11,12,13,14].

Recent advances in biological sex identification techniques include the genetic determination of sex using DNA extracted from skeletal remains [1]. Although molecular methods are more accurate, this approach is not free of technical limitations, as it is time-consuming and requires expensive equipment. Moreover, the efficiency of high-quality DNA retrieval from such methods may be affected by environmental contamination or the taphonomic status of bones [1,2].

Osteological-based sex estimation methods have been scrutinized to achieve standardization of methodologies and provide high levels of certainty when evaluating biological sex for legal decision making [20,21,22,23,24]. In addition to the repeatability, greater objectivity, and lower cognitive bias afforded by osteometric analysis of specimens, the most important factor inherent to this method’s validity is the classification accuracy. In some jurisdictions, the Daubert standard provides a set of criteria regarding the admissibility of expert witness testimony that includes the general acceptance of a method and demonstration of an estimable error rate [1,2,22]. In practice, methods of sex estimation are developing, and practitioners use the most accurate methodology available depending on the analyzed specimen and status of preservation [1,18,19,20,21].

Many studies recommend that the development of sex estimation methods should be based upon (1) a collection of a representative population dataset for model training and validation, (2) availability of biologically dimorphic skeletal traits, (3) satisfying the prerequisite statistical assumptions of the classification algorithms, and (4) presenting the probability of the concluded sex to reflect the uncertainty of diagnosis [3,4,9,10,17,18]. From a probabilistic standpoint, qualitative and quantitative sexing methods never reach the desirable 100% accuracy level [3,21,22]. When these markers are used in a binary decision system, each individual with a posterior probability (pp) greater than 0.5 of belonging to a specific group (in our case, sex) is classified in that group. Such sharp distinctions are not supported in the human skeleton, because skeletal measurements for each sex can have any figure in a spectrum of data [2]. This results in fluctuations in the rate of classification errors, as well as in the degree of SSD among the variables and skeletal elements other than the pelvis. Moreover, the level of activity and the population affinity are recognizable factors that influence the expression and pattern of sexual dimorphism [4]. Individuals on the extreme ends of phenotypic expression exhibit—on average—variable overall body size, producing an even larger amount of overlap between sexes [4], leading to a lower frequency of correctly classified individuals around the traditional dichotomizing point [10,16].

Several researchers investigated the pelvic and extra pelvic bones and tried to increase their accuracy by moving the posterior probability threshold for sex diagnosis up to 0.95, which resembles a magnitude of certitude that the given individual belongs to the given class [3,9,12,15,16,17,18]. For results based on good statistical evidence, they identified an indeterminate sex interval in which the posterior probability for male and female diagnosis overlaps. Applying such strict fixed criteria in practice does not guarantee that all models will reach this particular level of accuracy [11] due to the unavailability of analyzable skeletal specimens recognized for their high performance, i.e., pelvis. Consequently, some classification models may be significantly constrained due to their insufficient accuracy levels and/or the limited number of features in the classified specimens [3,9,12,13,15,16,17,18].

The selection of a posterior probability threshold slightly lower than 0.95 may be beneficial as proposed by several authors. Different studies have proposed that it must not be below 0.75 [8] or 0.80 [7,10,16,19]. The authors of these studies concluded that the threshold value to allocate sex should be cautiously tuned considering the posterior probability distributions and misclassification rates at each probability threshold. If the estimated posterior probabilities of individuals fall below these limits (i.e., <0.8 pp to >0.5), the threshold should be higher than this interval to maintain reasonable allocation accuracy [7]. In the case of higher posterior probabilities, the threshold might be lowered to increase the proportion of classified individuals without reducing the overall accuracy rate, albeit with unspecified error rates [7,8,25].

Jerković et al. [11,25] proposed the adjusted binary classification (ABC) method to investigate the individual posterior probability using any quantitative skeletal traits at which a particular (in presented cases 95%) level of global accuracy of sex estimates is achieved. This was accomplished by an algorithm written in the programming language R that excludes those individuals whose measurement values and/or prediction scores would classify them incorrectly (as the opposite sex). The results of their study also implied that the interval of undetermined individuals could be adjusted, such that the model could estimate the sex of a larger proportion of given specimens than a priori raising posterior probability levels to a fixed threshold while minimizing fluctuations in the model’s performance [11].

In this work, we are motivated by three related goals considered in the evaluation of statistical modeling and machine learning (ML) algorithms using the ABC approach: (1) comparing the predictive performance of different yet commonly used models at the traditional decision threshold and ABC-selected thresholds, (2) investigating the possibility of increasing their classification performance through the selection of the optimal sexing model and detection of the stability of performance after applying the ABC method on the holdout sample, and (3) identifying the ML algorithm that is best-suited for small training datasets. The outcomes of interest used to evaluate the possibilities and restrictions of the algorithms were the proportion of classified individuals (number of cases legible to classification) and each one’s overall accuracy, as well as cross-validated training data and test samples for each group.

## 2. Materials and Methods

### 2.1. Dataset

The data for the study comprised previously collected linear femoral measurements of a modern Turkish population [26], originating from 300 individuals (150 males and 150 females) with a mean age of 51 (58.01 ± 14.89 for males and 59.97 ± 13.85 for females). The measurements from the dataset were obtained from medical computed tomography (CT) scans of patients. Femora from images were reconstructed using the 3D Volume Rendering Technique (3D VRT) in OsiriX (v.5.6., Pixmeo, Geneva, Switzerland), and 13 femoral measurements were taken. The dataset [26] consisted of the following measurements: femur maximum length (FML) [23], femur bicondylar length (FBL) [24], femur trochanteric length (FTL) [24], vertical head diameter (VHD) [24], medio-lateral (transverse) midshaft diameter (MTD) [24], femur vertical diameter of neck (FVDN) [27], femur proximal breadth (FBP) [24], medio-lateral (transverse) subtrochanteric diameter (MLD) [24], epicondylar breadth (EB) [24], femoral bicondylar breadth (FBCB) [28], antero-posterior diameter of lateral condyle (APDLC) [24], and antero-posterior diameter of medial condyle (APDMC) [24]. The dataset did not specify the analyzed side.

### 2.2. Data Splitting

The initial sample (*n* = 300) was randomly split into a training and a testing subset with an equal proportion of male and female individuals. Accordingly, 80% of these data (*n* = 240) were assigned to the training set (120 males and 120 females), while the other 20% (*n* = 60) were assigned to the testing set (30 males and 30 females).

### 2.3. Validation Method

The discriminatory power of each variable and multivariable model was evaluated by cross-validation tests. We used the leave-group-out or Monte Carlo cross-validation algorithm (LGOCV or MCOCV). In this procedure, the initial training sample is randomly split into *n* calibration and validation subsamples. The calibration set is used for model development, while the validation sample is used to test the model’s performance on data that were not used to develop the model [29]. We used 70% of the data for the calibration set, while 30% were used for validation. This procedure was iterated 50 times for each model, and classification performance metrics were calculated as the average of all iterations. This cross-validation technique was selected because it tends to produce estimates of the error rate with smaller variance than the leave-one-out methodology [30].

### 2.4. Statistical Analysis, Modeling, and Visualization

Statistical analyses, modeling, and visualization were performed in R statistical software version 3.6.2 and RStudio version 1.2.1335 [31,32] with packages caret, dplyr, ggpubr, lattice, MachineShop, mlbench, overlapping, patchwork, and tidyverse [31,32,33,34,35,36,37,38,39,40]. All statistical analyses were conducted with the significance level set at *p* ≤ 0.05.

#### 2.4.1. Sexual Dimorphism

We calculated basic descriptive statistical parameters for all variables for males and females, including the mean, standard deviation, and range. To examine sexual dimorphism, we used an independent-sample *t*-test. In the last step, we estimated the overlap between male and female measurements through the overlap in their kernel density estimates. The overlap was expressed using an index that indicates the percentage overlap between estimated kernel densities [38].

#### 2.4.2. Feature Selection

##### Univariate Feature Selection 

From 13 variables, we selected the five best variables by ranking the features according to their importance using the varImp function on a cross-validated dataset. The variable importance was assessed by a receiver operating characteristic (ROC) curve analysis that was conducted for each predictor. In this procedure, different cutoffs were used to classify specimens into a group using the selected variable. In the next step, the algorithm estimates the sensitivity and specificity for each cutoff and computes the area under the ROC curve using the trapezoidal rule [31,41].

##### Multivariate Feature Selection

We selected the optimal set of features for the multivariate models on cross-validated data using the recursive feature elimination (RFE) algorithm from the mlbench package [37]. The RFE algorithm recursively removes the predictors and constructs classification models using the remaining variables. By considering classification accuracy, the algorithm estimates the optimal number of variables and the set of variables that most contribute to the classification [40,41]. In the present study, RFE was conducted for each classifier, considering all 13 variables and their combinations.

#### 2.4.3. Fitting the Classification Models

We developed univariate sex classification models for five variables that showed the greatest importance according to ROC curve analysis and one multivariate model that included variables selected by the RFE algorithm. To develop models, we employed the train function inside the caret package and selected the methods from a list of available models. LGOCV was incorporated with the trainControl function [31]. We generated posterior probabilities of class membership for each model, for both cross-validated and test datasets. In the present study, we used four linear classification techniques: logistic regression (LR), linear discriminant analysis (LDA), boosted generalized linear model (GLMB), and support vector machine with a linear kernel (SVM). LR and LDA were selected as they are the most common algorithms for sex/ancestry classification in biological and forensic anthropology [4,8,9], while SVM was chosen due to it being a broadly used ML model that also proved to be efficient in osteometric sex classification [42,43]. GLMB was employed as an adopted technique for increasing the accuracy of standard linear models [44].

##### Logistic Regression

Logistic regression examines the relationship of predictive variables with a binary outcome. It develops a linear model that predicts a transformation of the outcome variable—the logit function. The logit function, which is the natural log of the odds that a specimen belongs to one of the classes, is then used to calculate the posterior probability that the specimen belongs to the first or second class. Classification is usually conducted by assigning a specimen to a group with the highest probability [9,45]. We used the glm method (family = binomial) with no tuning parameters to develop the LR model.

##### Linear Discriminant Analysis

Linear discriminant analysis explores a linear combination of variables and creates a discriminant function that can discriminate mutually exclusive groups [46,47]. Although the LDA is today mainly considered a dimensionality reduction technique, it works efficiently as a linear classifier [47]. In the latter case, LDA uses a discriminant function score and classifies specimens into the group whose centroid is the closest to the score [46,47]. Posterior probabilities are calculated by the Bayes theorem. We used the LDA method for classification from package MASS (with no tuning parameters) to develop the LDA model [48].

##### Boosted Generalized Linear Model

The boosted generalized linear model is an adapted standard linear model designed to reduce bias using a boosting algorithm. This modeling technique trains data using the best turning parameter (mstop) in the validation sample and conducts variable selection. If early stopping is used, the effects can be shrunken toward zero. For each boosting iteration, the algorithm fits linear models for each column of the design matrix to the negative gradient vector utilizing the only best-fitting model in the update step [49,50,51]. To develop the GLMB model, we used the glmboost method for classification or regression using packages plyr and mboost with tuning parameters (number of boosting iterations and pruning). Class posterior probabilities were calculated by minimizing the negative binomial log-likelihood [51]. We used default settings for tuning, with no pruning, and the number of boosting iterations (mstop) was set at 50, 100, and 150.

##### Support Vector Machine with Linear Kernel

Support vector machine is also a method from the family generalized linear models for prediction and classification using a linear combination of variables [52]. In the case of classification, this algorithm searches for a hyperplane that best separates two classes by considering the “sum of the distances from the hyperplane to the closest positive and negative correctly classified samples” [46]. If the hyperplane can be located in the original data instead of higher-dimensional space, we implement an SVM with a linear kernel [52]. To develop the SVM model, we used the method svmLinear that performs classification or regression using package kernlab with tuning parameters (cost, C). The posterior probabilities were obtained using the modified Platt’s method [9,53,54]. We used default settings for tuning, with parameter “C” held constant at a value of 1.

#### 2.4.4. Classification Performance Metrics

In binary classification, there are only two classes, which are usually referred to as positive and negative [2,6,14]. This allows for two cases of misclassification: false negative (predicting negative when the actual class is positive) and false positive (predicting positive when the actual class is negative), and both are of equal importance when dealing with forensic contexts [2,13,14]. To evaluate classification models, we constructed confusion matrices and calculated the number of true positives (TP), false positives (FP), true negatives (TN), and false negatives (FN). The male sex was marked as positive (P), while the female sex was labeled as a negative class (N). Using these parameters, we calculated the basic evaluation metrics in a cross-validated and testing set: accuracy, sensitivity, specificity, positive predicted value (PPV), and negative predicted value (NPV).

Additionally, we calculated the concordance index or c-index, which measures the discriminatory power of the model [55]. This score shows whether the model will provide a higher probability for the actual group in which a specimen belongs, among the randomly chosen pair of specimens, one belonging to the first and one to the second group. The values of the c-index range from 0.5 to 1, where a lower value indicates a model with no discriminatory ability, and the highest value shows perfect discrimination [15,16]. For each classification model, we also calculated the overlap degree between posterior probabilities of male and female specimens, with the same approach described in Section 2.4.1.

#### 2.4.5. Adjusted Binary Classification (ABC) Algorithm

The adjusted binary classification algorithm was conducted to find posterior probability cutoffs that exclude specimens in the overlapping area and provide a classification accuracy, PPV, and NPV of at least 95% [25]. Using the R-code based on the algorithm from the study by Jerković et al. (2021), we calculated posterior probability thresholds for each classification model in the cross-validated dataset and computed the proportion of the specimens that the model could classify [25]. We used developed thresholds to classify the sex of specimens in the testing dataset, and we calculated the proportion of classifiable specimens and the classification metrics.

### 2.5. Assessing the Sample Size Effect

To examine the influence of the training set size on the classification performance in the independent (test) sample, we randomly generated different sample sizes and used them to develop classification models. The sample size conditions specified in this study included 19 samples, ranging from 10% (*n* = 24) to 100% (*n* = 240) of the original training sample, with an increment of 5%. For each sample size and classifier, we used the RFE algorithm to obtain the optimal number and combination of variables.

## 3. Results

### 3.1. Sexual Dimorphism

Table 1 presents the descriptive statistics for each metric variable, overlap index, and *t*-test results in the training dataset (*n* = 240). Statistically significant differences were found between males and females in all variables (*p* < 0.001). The overlap index was highest for the diaphyseal width measurements MTD and MLD, moderate for the length measurements FML, FBL, and FTL, and low for the epiphyseal measurements. The most sexually dimorphic variable was FEB, followed by VHD, FVDN, and FNAL.

### 3.2. Classification Performances for Traditional pp Cutoff (0.5) in LGOCV Sample

Table 2 depicts the average values of the overall accuracies, sensitivity, specificity, PPV, and NPV derived from the cross-validation analysis (LGOCV) for the LR, LDA, GLMB, and SVM models using the 0.5 posterior probability cutoff. The performance of the four modeling techniques was consistently comparable with differences in the overall accuracy rate by the decimal points. For the univariate models, FVND yielded the highest overall accuracies between 86.33% and 86.50%. FEB and VHD achieved accuracies between 84.36% and 84.89%, FBCB achieved accuracies between 83% and 83.94%, and FNAL achieved accuracies between 82.19% and 82.25%. The multivariate models yielded the highest accuracies (89.17–90.08%).

None of the models reached 95% global accuracy or 95% PPV and NPV. According to the C-index, the discriminant power of the classifiers was perfect (greater than 0.90) for all the variables and classifiers. The variables selected as the best predictors for the four classification methods are presented in the footnote of Table 2.

### 3.3. Application of the ABC Approach in the Training Sample

Table 3 shows the posterior probability cutoff values for males and females computed with the ABC algorithm to classify sex with the PPV/NPV ≥95% criteria. In univariate models, these values ranged from 0.625 to 0.930 (for the FEB and FBCB) in males and from 0.806 to 0.999 (for FBCB and FVND) in females, allowing us to overall classify between 28% and 87% of individuals. In the multivariate models, pp thresholds were remarkably lower and ranged from 0.639 to 0.734 (for the GLMB and LR) in males and from 0.778 to 0.867 (for LR and LDA) in females. The proportion of classifiable individuals increased to 81.94–85.58% with a small sexing bias between 0.7% for LR and 7.9% for GLMB.

The accuracy was above 95% independent of the considered variables and ML algorithm, but the female classification accuracy (specificity) showed more variability across the different femoral measurements and algorithms (Table 3). In contrast to univariate models, all multivariate models achieved stable classification results (around 95%) for PPV and NPV, as well as for sensitivity and specificity. Close-to-perfect c-index values (ranging between 0.975 to 0.985) were observed for all models across all studied variables in the simple and multiple variable equations, except for the VHD and FVND.

The classifiers produced different subsets of best predictors in terms of the number and type, but three features were common to all the selected attribute sets (FVDN, FEB, and FNAL). LR led to accurate results with much fewer variables than the other three models with greater group separation, i.e., more individuals were classified closer to 0 and 1 probabilities. Both LDA and SVM produced the same subset of features. The GLMB algorithm showed the highest discriminatory performance in the training data for the univariate models after applying the ABC approach. More detailed performance indicators analyzed by the posterior probabilities are shown in Supplementary Figure S1.

### 3.4. Model Performance in the Testing Sample

Table 4 reveals that all models produced stable comparable results in the test sample. All the algorithms demonstrated outstanding performance for this dataset using the c-index. For the multivariate model, LDA and SVM had the highest values with near-perfect c-index values of 0.990 and 0.991, respectively.

The PPVs and NPVs peaked above 95% in almost all models, but the FBCB was just below this level. The number of individuals classified by all the univariate models was identical in all variables except for VHD in LDA, FVND in GLMB, and FNAL in LR and SVM. The proportion of classified individuals ranged from 36.66% to 90% in males and 0% to 93.33% in females. The highest proportion of classified individuals in both sexes was obtained using the multivariate function. In terms of model performance judged from the proportion of classified individuals, GLMB and SVM ranked the highest amongst the classifiers tested. The differences added by SVM were trivial in comparison to GLMB with regard to the other performance metrics. For example, SVM increased the proportion of classified males using the FNAL model by only 3.33%, corresponding to the only misclassified male as female, but this reduced the sensitivity and NPV to 92.3% and 95.24%, respectively. On the basis of the performance parameters mentioned above, the best classifier was GLMB, and the best single variable was FNAL (proximal end of the femur) followed by FBCB (distal end of the femur), as well as the multivariate model, which included a different set of variables according to the type of classifier. Case-wise classification results are visible in Supplementary Figure S2.

### 3.5. Sample Size Effect

**Figure 1:**
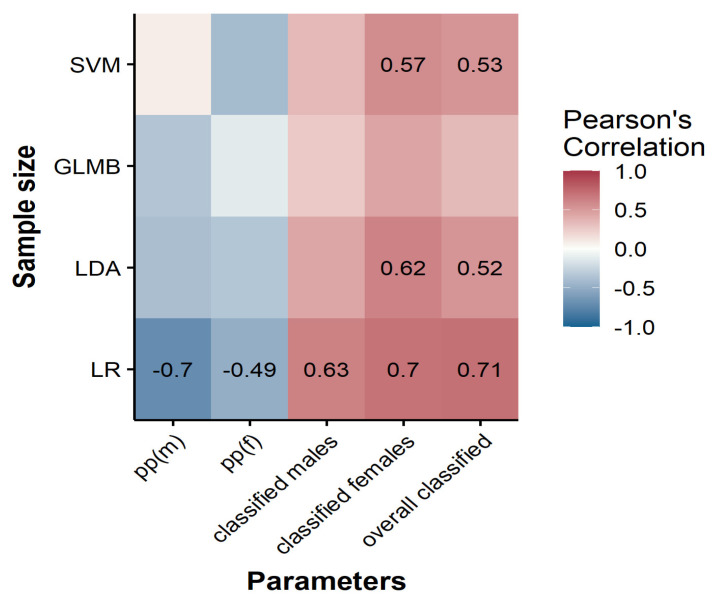
Correlation plot between the sample size and its effect on pp cutoff values and the percentage of classified males and females, as well as the overall classified individuals. Correlation coefficients are shown for statically significant values only at *p* < 0.05.

Figure 1 demonstrates a moderate to high correlation (0.489 to 0.708) of the endpoint parameters (the pp level and the number of classifiable individuals) with the sample size. A negative correlation between the pp thresholds and the sample size was observed only in LR, meaning that increasing the sample size was associated with lowering the pp thresholds and increasing the number of classifiable individuals. Selecting a pp threshold was not shown to be dependent upon the sample size for LDA and SVM, but increasing the sample size was positively correlated with the number of classifiable individuals in the female group and, subsequently, the overall sample. GLMB was the only classier that showed stability in the number of classifiable females regardless of the sample size.

**Figure 2:**
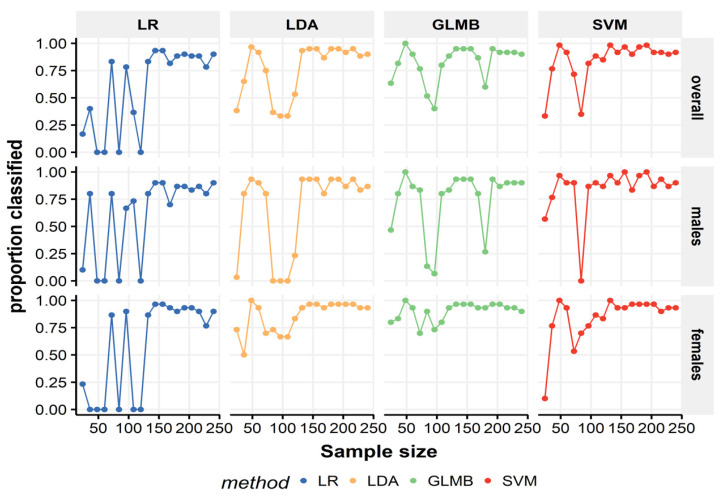
Influence of the training sample size on the proportion of classified specimens in the testing sample. Several sample sizes of the training dataset are plotted against the proportion of classified individuals (overall and sex specific rates) in the test dataset (*n* = 60) using different ML models; each algorithm has its own panel.

Figure 2 presents the proportion of classified individuals in the test sample when models were constructed using different training sample sizes. The impact of going from small to larger sample sizes was the greatest on LR for both sex groups. For other classifiers, the male group was the most affected, particularly with sample sizes of 96 and 84. Under a small sample size (*n* < 100), LR performed worse than LDA at 20% to 30% of the training sample (i.e., *n* = 48, 60, and 72, respectively). SVM and GLMB had comparable results for the classifiable proportions (males, females, and overall), but SVM performed slightly worse when the sample size was >100. Even though the LR performed worse than the other algorithms with small datasets, as the number of cases increased to >150, LR showed a substantial performance improvement.

**Figure 3:**
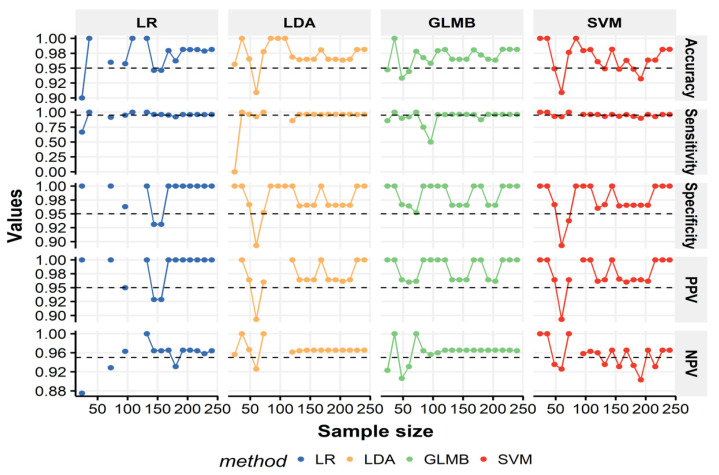
Influence of the training sample size on the classification performance parameters in the testing sample (*n* = 60). It should be noted that multivariate models were computed using the variable selection procedure. The interrupted line delineates the desired level of 95%.

In Figure 3, we can observe a fluctuation in the average performance parameters of the various models as a function of sample size, particularly the LR model. The impact of sample size was consistent across the LDA and SVM models. The overall classification error was 10% to 100% for models with fewer than 100 specimens. It decreased to ~2% and 5% as the sample size increased to or above 150, with a sharp rise in the total and sex-specific number of classified individuals and stabilized performance measures (above 95%). The negligible differences were observed between the models when the sample size increased above 150. A sample size between 100 and 150 data points or more was convenient to reach the required level of PPV and NPV for the tested models.

While the proportion of classified individuals increased, PPVs and NPVs showed more considerable variations, but all above the 95% cutoff limit. The exception occurred for SVM in 80% and 90% of the training sample (*n* = 192 and 216), where the accuracy, sensitivity, and NPV were <95%, although SVM achieved the highest rate of classifiable individuals at 80% of the training size. PPVs and NPVs for SVM reached 95% at a sample size of 10% to 15%, i.e., below 50 data points; however, beyond this threshold, PPV again decreased to 92%. A decrease in both NPV and total accuracy was evident in SVM due to the lowest sensitivity rates obtained with these sample sizes. Both GLMB and LDA provided similar accuracy rates under varying sample sizes. GLMB slightly outperformed LDA overall, notably in examples with sample sizes <100 (Figure 3). GLMB was superior to other algorithms for any volume of training data. Its accuracy started at 95% for 10% of the data and peaked at above 98% when the training data increased to the whole training dataset. None of the learning models other than GLMB could achieve acceptable results when using 35% of the dataset (*n* = 84), and they could not classify any of the males in the test sample. Supplementary Material, Spreadsheet demonstrates the performance indicators at each sample size of the training data using the different classification techniques.

## 4. Discussion

The presented results showed that the ABC approach is a reliable procedure to estimate sex using femoral measurements in the analyzed population sample with a very high accuracy level, PPV, and NPV (>95%). The overall classification models’ performance was generally not dependent on the type of the ML algorithm applied when the full training sample size was employed. Sex bias was noted in the proportion of classified specimens, as well as in the classification performances in univariate models. When we applied multivariate models, the named bias was minimized, and sex could be estimated in more than 80% of individuals in cross-validation and 90% in the testing sample, with accuracy greater than 95%. The results demonstrate that performance using different machine learning methods within the ABC framework can be differently affected by the size of the training sample, which should be considered when developing osteometric sex classification standards. Forensic anthropologists could, therefore, apply the ABC method to construct sex estimation standards when the level of accuracy obtained by traditional thresholding is not sufficiently high for classification in forensic settings.

### 4.1. Sex Estimation Using the Default Cutoff Threshold of 0.5

All 13 femoral variables showed pronounced and statistically significant sexual dimorphism with more marked expression in joint surfaces than the shaft measurements, which is also a common observation in similar studies [10,11,12,14,15]. From these initial set of 13 variables selected, only five features were included in the univariate trait analysis due to their consistently high sexual dimorphism indicators, as demonstrated by the ROC curve. These variables showed a degree of accuracy in the CV sample of 82.3–87.4% for univariate and 91.6–92.9% for multivariate models. None of them met the accuracy criteria set by the present study (95%), which was the case in the most previous studies that achieved accuracies between 60% to 87.5% in the univariate models and from 84% to 92.5% in the multivariate models [10,11,12,13,14,26], even after employing ML methods [13,14].

The current study detected almost no or only slight differences (from 0.06% to 0.94%) between the ML classifiers (LR, LDA, GLMB, and SVM) when the total training sample with an equal sex ratio was analyzed at the 0.5 decision threshold. Similar findings were also noted previously by several studies [14,42,43]. For example, Curate et al. [14] did not detect significant differences in accuracy when estimating sex using femora with four classifiers LR, LDA, SVM, and reduced error pruning tree. Toneva et al. [43] showed a similar performance in sex estimation on cranial measurements when using LR and SVM, while Nikita and Nikitas [42] also confirmed that both LDA and SVM linear algorithms could be considered efficient when dealing with sex/ancestry classification using continuous variables.

### 4.2. Sex Estimation Using ABC Approach

We used the posterior probability scale with two different classification rules—one for each sex that accounts for the “zone of uncertainty” in each variable. From the initial accuracy rates that did not exceed 87% for univariate and 90% for multivariate models, we managed to raise accuracies to 95% or higher by applying the ABC algorithm. Such accuracy was achieved by all models in the CV sample, while only one univariate model (FEB) failed to meet that level. Losses in terms of the overall proportion of specimens left “indeterminate” ranges for univariate models between 24.8% and 72% in CV and 18.3% and 70% in testing sample. They were remarkably lower in the multivariate models and ranged from 13.4% and 19.3% in CV and 8.3% and 10% in the testing dataset. The variations in applied ML classifiers in the training and test samples were also minor, in terms of both the classification performance and the proportion of classified specimens. The overall results concurred with the original proposal of the ABC method, whereby, using LDA to classify sex from handprint measurements, the authors obtained a proportion of unclassified specimens of 23–71% for univariate and 12–13% for multivariate models with accuracies above 95% [25]. In contrast to the examined ABC approach, other studies that raised the pp thresholds (0.80, 0.90, and 0.95) to embrace the concept of the trichotomic approach left a large number of individuals unclassified [3,9,12,15,18,25].

The unclassified individuals in test samples of univariate and multivariate models ranged between 53.5–85.7% and 39.1–50.9% for the humerus [15], between 60.3–83.3% and 37.3–66.7% for the femur [12], and between 70% and 93% for the multivariate cranial models [9] at pp > 0.95 depending on the available skeletal element, the type and number of variables in the models, and the population sample under study.

### 4.3. The Training Sample Size Effect on the Performance of Multivariable Models

The number of observations in the training set and the classification rates were correlated, agreeing with the understanding that the models’ generalizability improves with the feeding of more data points [56,57]. Unlike the almost equal performance of different ML classifiers in the full training sample size, remarkable differences were observed when we simulated different sample sizes of the training datasets to generate multivariate models. The most robust model for limited metric data was GLMB, followed by LDA and then SVM, while the least robust model was LR. In cases of a small training sample size, the calculated pp for both males and females was very high, approximately 1.0 (Supplemental Materials, Spreadsheet of the performance indicators at each sample size of the training data using the different classification techniques).

Our findings suggest that LR may not be the best method for developing sex classification standards with limited sample sizes, and that LDA could be a more robust classifier with small samples, in agreement with the findings of previous studies [58,59]. The performance of LR can be explained by its parameters calculated using the iterative maximum likelihood estimation (MLE), which requires a large sample size to obtain estimates. The performance instability of the LR with small sample sizes can be attributed to the absence of MLE of one or more coefficients of the explanatory variables which take values of plus or minus infinity, because of the “separation effect” associated with sampling artefacts, as well as the severely unequal rates of classifiable cases after applying the ABC approach [60].

Therefore, the study did not find elements to support the suggestions of Bartholdy et al. [16] who recommended the use of LR over LDA due to less stringent model assumptions and the potential to provide probabilities instead of dichotomous results. Moreover, LR and LDA methods only differ in the way they estimate the coefficients, but the same formula can be used to derive the pp for both LR and LDA models when the values of α (constant) and β (coefficient) are known [18,59]. With regard to the other ML methods, the complexity underlying their computation hinder the application of this formula [42]. The core design of the current study differed from that presented by Bartholdy et al. [16] in several experimental factors such as (1) the stratification of sample size from the small to the moderately large sample, (2) the higher number of features analyzed, and (3) the proportion of classified individuals recorded and compared at different cutoffs on the probability scale. Although their results indicated that both classifiers produced consistent accuracies, they concluded that LR is better than LDA and supported their conclusions with studies based on different data types (i.e., nonmetric morphological data with ordinal scores) which often violate the normal distribution assumption.

Among nonstandard algorithms, GLMB showed the most stable performance, almost independently of the sample size and when there were more variables than observations. This observation was also described by Tutz and Binder [44]. Considering GLMB’s simplicity and performance, the traditional LDA does not rank much lower than GLMB and SVM [42]. SVM could be a practical ML alternative to LDA when an adequate sample size is available because the SVM method requires tuning of a single hyperparameter [13,42]. These results imply that the ABC approach could also be applicable with a relatively small sample size training set, which could be helpful not only in forensics, but also in bioarcheological contexts.

### 4.4. Study Limitations and Future Directions

Although the ABC approach showed overall excellent performance in the presented osteometric sex estimation case, we consider several shortfalls. When there are substantial differences between the posterior probability distributions in the training and testing datasets, the method’s performance can be hampered despite fulfilling the equal prior assumptions in both datasets [21]. This can be illustrated with the case of SVM at the sample size (*n* = 192), where we achieved the highest number of classifiable individuals at their calculated pp thresholds, but with accuracy, sensitivity, and NPV below 95% (see also Supplementary Materials, Spreadsheet of the performance indicators at each sample size of the training data using the different classification techniques).

Comparison of the magnitude of sexual dimorphism among skeletal traits or worldwide populations has to consider possible differences in degrees of closeness to the opposite sex distribution, either because of different distances between mean values or, even in the case of equal distances, because of different extents to and densities of the intrasexual variability [61]. The overall performance of classifiers on the unseen data depends on the extent to which a test dataset represents the original distribution rather than its size because the ML model is constructed to best describe the distribution and structure pattern in the training data [57]. Differences in the probability distribution (covariate shift) [13,62] between the training and test samples should also be considered because it may lead to deterioration of the model performance on the independent population sample as the model is not pretrained on the degree of overlap between distributions of males and females in the new test sample [13].

One of the potential drawbacks is that the ABC adjustment of pp thresholds for males and females can be imbalanced in univariate models, leading to considerably different rates of classifiable individuals per sex (low or high). If the classified groups are extremely unequal in size, the misclassification rate for the smaller ones will be very high regardless of the classification method. The PPVs and NPVs were, thus, lower in models with lower classifiable instances rates and could increase more rapidly with increasing specificity than with increasing sensitivity. A greater overlap between groups results in a larger disparity in the pp thresholds with unequal rates of classifiable individuals, and a lower PPV and NPV can be expected [9,18,63,64]. However, the most important finding is that those “imbalances” in the number of classifiable individuals can be almost ruled out by considering multiple predictors [25].

We also consider that we employed the default tuning parameters because controversy still prevails regarding the effects of different conditions of hyperparameter tuning on classifier performance, as well as the different methods of variable selection [42]. The “statistical opportunism” effect can lead to overfitting, which is problematic because the same variables may not be selected uniformly by each classification technique and/or sample size, even those with the strongest sexual dimorphism [9]. Previous studies with different skeletal elements datasets (cranial and/or postcranial bones) did not agree on the classifier that ranked best in all the classification applications such as sex and ancestry estimation, e.g., the studies by Nikita and Nikitas [42] and Santos et al. [9]. Future research should focus on the difference between the ML techniques in calculating the posterior probabilities of data when there is an extreme subclass imbalance with different priors for forensic and archeological applications.

Lastly, it should be emphasized that the application of the ML methods to CT images by considering the manually taken linear measurements does not take full advantage of available computation methods. For example, modern approaches employ deep learning techniques and convolutional neural networks that can be adjusted to directly estimate sex from images without taking measurements or placing landmarks [64,65]. Thus, principles contained in the ABC approach can also be tested in more advanced contexts to additionally improve classification accuracy when required.

## 5. Conclusions

Compared to the traditional classification approach using fixed pp thresholds, i.e., 0.5 and 0.95, the ABC achieves remarkably higher accuracy in a relatively large proportion of classifiable individuals while adjusting the precision of the classification at a 95% level. The ABC approach is an “uncertainty-aware framework”, allowing customized posterior probability computations to find an acceptable classification performance across different sample sizes while controlling for PPV and NPV, but not the sensitivity and specificity rates.

The results presented here reveal that, in sex classification, dataset size is not necessarily an obstacle to compute a high-performing model since the average performance of classifiers reached 100% on some small datasets and the generalizability of the model on test data depended on accurate estimates of the moments of the training sample distribution. We also studied the pp distribution of each variable, showing their effect on the performance of different traits regardless of the model used.

The efficiency and reliability of the ABC approach for estimating sex were most apparent when (1) the sample size was large enough with *n* > 100 (>150 for LR only), (2) the variables were sexually dimorphic with minimal overlap between male and female distributions, (3) the posterior probability cutoffs for each sex were approximately balanced, and (4) the multivariate models were used to overcome the imbalances in the classifiable proportions of individuals.

## Figures and Tables

**Table 1:** Descriptive statistics with *t*-test results and overlapping percentages in the training sample.

Variables	Males (*n* = 120)		Females (*n* = 120)		*t*-Test		Overlapping
	Mean ± SD (mm)	Range (mm)	Mean ± SD (mm)	Range (mm)	*t*	*p*	(%)
FML	443.78 ± 25.59	384.70–502.30	406.51 ± 21.20	359.24–453.13	12.289	<0.001 *	27.34
FBL	441.23 ± 25.76	382.12–501.03	404.73 ± 21.92	358.61–453.17	11.821	<0.001 *	28.52
FTL	425.65± 23.77	371.24–477.58	391.19 ± 21.05	342.70–439.45	11.890	<0.001 *	26.55
MTD	29.33 ± 2.20	22–34.14	28.09 ± 2.37	21.88–37.10	4.205	<0.001 *	63.39
VHD	49.19 ± 3.02	38.89–55.77	43.19 ± 2.89	36.48–50.84	15.715	<0.001 *	19.57
FVDN	36.75 ± 2.69	27.85–43.25	31.98 ± 2.24	25.77–37.47	14.916	<0.001 *	17.56
FNAL	102.20 ± 6.27	87.23–122.38	91.14 ± 5.36	79.92–103.27	14.682	<0.001 *	22.51
FBP	91.28 ± 5.88	71.49–108.42	81.61 ± 5.08	72.06–94.30	17.908	<0.001 *	23.00
MLD	32.95 ± 2.44	26.32–39.51	31.07 ± 2.24	26.16–37.41	6.231	<0.001 *	45.78
FBCB	74.71 ± 4.31	65.10–89.03	66.61 ± 4.19	58.78–76.38	14.777	<0.001 *	23.70
FEB	85.72 ± 4.42	76.46–98.06	76.70 ± 3.58	67.86–89.58	17.908	<0.001 *	17.15
APDLC	64.23 ± 3.67	53.58–73.84	58.32 ± 3.22	51.38–67.91	13.267	<0.001 *	23.82
APDMC	63.54 ± 3.70	53.97–73.21	57.79 ± 3.54	49.84–67.40	12.304	<0.001 *	28.69

* *p* < 0.05 was considered significant.

**Table 2:** LGOCV classification results without employing the ABC approach in the training sample.

Variables	Accuracy (%)	Sensitivity (%)	Specificity (%)	PPV (%)	NPV (%)	c-Index
Logistic regression						
FEB	84.44	85.89	83	83.48	85.47	0.944
VHD	84.36	85.06	83.67	83.89	84.85	0.920
FVDN	86.33	85.44	87.22	86.99	85.70	0.914
FBCB	83	81.22	84.78	84.22	81.87	0.904
FNAL	82.25	83.17	81.33	81.67	82.85	0.903
LR1	90.08	87.67	92.50	92.12	88.24	0.968
Discriminant analysis						
FEB	84.89	85.28	84.50	84.62	85.16	0.944
VHD	84.72	86.44	83	83.57	85.96	0.920
FVDN	86.42	85.44	87.39	87.14	85.72	0.915
FBCB	83.06	81	85.11	84.47	81.75	0.905
FNAL	82.19	81.78	82.61	82.46	81.93	0.904
DF1	89.58	86.22	92.94	82.44	87.09	0.959
Boosted glm						
FEB	84.89	85.28	84.50	84.62	85.16	0.945
VHD	84.69	86.64	82.94	83.52	85.95	0.920
FVDN	86.42	85.44	87.39	87.14	85.72	0.914
FBCB	83.94	82.89	85	84.68	83.24	0.906
FNAL	82.19	81.78	82.61	82.46	81.93	0.904
GLMB1	89.17	86	92.33	91.81	86.83	0.961
SVM linear						
FEB	84.50	85.83	83.17	83.60	85.45	0.944
VHD	84.42	85.28	83.56	83.83	85.02	0.919
FVDN	86.50	85.61	87.39	87.16	85.86	0.914
FBCB	83.06	81.33	84.78	84.23	81.95	0.904
FNAL	82.22	83.17	81.28	81.62	82.84	0.904
SVM1	89.78	88	91.56	91.24	88.41	0.958

Multivariate feature selection: LR: FVDN + FEB + FNAL + MLD; LDA: FEB + VHD + FVDN + FBCB + FNAL; boosted GLM: FEB + FVDN + VHD + MLD + MTD + FNAL + FBCB + APDLC + FML + FBP + APDMC; SVM: FEB + VHD + FVDN + FBCB + FNAL.

**Table 3:** LGOCV classification results with ABC adjustment in the training sample.

Variables	Posterior Probability Cutoff		% of Classified Cases			Accuracy (%)	Sensitivity (%)	Specificity (%)	PPV (%)	NPV (%)	c-Index
	Males	Females	Males	Females	Overall						
Logistic regression											
FEB	0.691	0.883	82.05	65.94	71.94	95.08	96.14	93.77	95.05	95.13	0.978
VHD	0.741	0.994	68.28	9.50	38.89	95.21	99.76	62.57	95.04	97.27	0.898
FVDN	0.837	0.999	55.83	3	28	95.09	100	3.70	95	100	0.872
FBCB	0.930	0.854	33.78	51.56	42.58	95.17	92.40	96.98	95.23	95.14	0.984
FNAL	0.832	0.866	48.50	53.44	50.97	95.03	94.50	95.52	95.05	95.02	0.981
LR1	0.734	0.778	86.94	86.22	86.58	95.03	95.08	94.97	95.02	95.04	0.980
Discriminant analysis											
FEB	0.669	0.899	82.5	67.89	75.18	95.05	96.03	93.86	95.00	95.11	0.977
VHD	0.794	0.991	68.61	11.78	40.19	95.09	99.43	69.81	95.05	95.48	0.920
FVDN	0.838	0.999	56.28	3	29.64	95.13	100	3.70	95.11	100	0.894
FBCB	0.928	0.854	34.17	52.22	43.19	95.31	92.36	97.23	95.62	95.11	0.984
FNAL	0.880	0.870	48.72	53.89	51.31	95.13	94.53	95.67	95.18	95.08	0.981
DF1	0.726	0.867	84.39	79.94	82.17	95.03	95.33	94.72	95.01	95.05	0.977
Boosted glm											
FEB	0.625	0.830	82.39	67.83	75.11	95.04	95.95	93.93	95.06	95.02	0.978
VHD	0.705	0.975	68.67	12.67	40.67	95.22	99.51	71.93	95.05	96.47	0.926
FVDN	0.794	0.993	56.5	4.11	30.31	95.14	100	28.38	95.05	100	0.918
FBCB	0.885	0.806	39.11	53.50	46.31	95.20	93.32	96.57	95.22	95.19	0.985
FNAL	0.848	0.839	48.44	52.44	50.44	95.15	94.84	95.44	95.06	95.24	0.982
BGLM1	0.639	0.815	85.89	78	81.94	95.05	95.54	94.52	95.05	95.06	0.979
SVM linear											
FEB	0.672	0.864	81.89	66.06	73.97	95.08	96.07	93.86	95.10	95.06	0.977
VHD	0.723	0.990	68.22	10.06	39.14	95.03	99.51	64.64	95.02	95.12	0.908
FVDN	0.830	0.998	54.33	2.94	28.64	95.34	100	9	95.32	100	0.903
FBCB	0.915	0.839	34.22	52.17	43.19	95.11	92.37	96.91	95.15	95.09	0.983
FNAL	0.871	0.849	48.78	52.39	50.58	95.17	94.76	95.55	95.19	95.14	0.981
SVM1	0.719	0.783	82.55	78.78	80.67	95.04	95.29	94.78	95.03	95.05	0.975

**Table 4:** Classification results in the testing sample with ABC adjustment (*n* = 60).

Variables	% of Classified Cases			Accuracy (%)	Sensitivity (%)	Specificity (%)	PPV (%)	NPV (%)	c-Index
	Males	Females	Overall						
Logistic regression									
FEB	80	83.33	81.67	91.84	95.83	88.00	88.46	95.65	0.987
VHD	63.33	13.33	38.33	100	100	100	100	100	1
FVDN	60	0	30	100	100	/	/	/	/
FBCB	40	53.33	46.67	96.43	91.67	100	100	94.12	0.953
FNAL	36.66	66.67	53	100	100	100	100	100	1
LR1	90	90	90	98.15	96.30	100	100	96.43	0.989
Discriminant analysis									
FEB	80	83.33	81.67	91.84	95.83	88.00	88.46	95.65	0.987
VHD	66.33	13.33	38.33	100	100	100	100	100	1
FVDN	60	0	30	100	100	/	/	/	/
FBCB	40	53.33	46.67	96.43	91.67	100	100	94.12	0.953
FNAL	40	66.67	53.33	100	100	100	100	100	1
DF1	86.67	93.33	90	98.15	96.15	100	100	96.55	0.990
Boosted glm									
FEB	80	83.33	81.67	91.84	95.83	88	88.46	95.65	0.987
VHD	63.33	13.33	38.33	100	100	100	100	100	1
FVDN	63.33	0	31.67	100	100	/	/	/	/
FBCB	40	60	50	96.67	91.67	100	100	94.74	0.951
FNAL	40	66.67	53.33	100	100	100	100	100	1
BGLM1	90	90	90	98.15	96.30	100	100	96.43	0.989
SVM Linear									
FEB	80	83.33	81.67	91.84	95.83	88	88.46	95.65	0.987
VHD	63.33	13.33	38.33	100	100	100	100	100	1
FVDN	60	0	30	100	100	/	/	/	/
FBCB	40	53.33	46.67	96.43	91.67	100	100	94.12	0.953
FNAL	43.33	66.67	55	96.67	92.31	100	100	95.24	1
SVM1	90	93.33	91.67	98.18	96.30	100	100	96.55	0.991

## Data Availability

Turkish data were obtained from the PhD thesis of O. Gulhan and are available at Cranfield University, UK (Skeletal sexing standards of human remains in Turkey. Available online: https://dspace.lib.cranfield.ac.uk/bitstream/handle/1826/12272/O%20Gulhan%20PhD.pdf?sequence=1 (accessed on 24 April 2020). The permission for use was secured from Prof. Andrew Shortland (personal communication) prior to data analysis.

## Supplementary Materials

The following supporting information can be downloaded at https://www.mdpi.com/article/10.3390/biology11060917/s1: Spreadsheet of the performance indicators at each sample size of the training data using the different classification techniques. Figure S1, Comparison between the estimated posterior classification probability densities of the two sex groups in the training set; Figure S2, Plot of the posterior probabilities (pp) values as a histogram in terms of individual frequencies.

## References

[1] Ubelaker D.H., Katzenberg A., Grauer A.L. (2018). Forensic anthropology: Methodology and applications. Biological Anthropology of the Human Skeleton.

[2] Klepinger L.L. (2006). Fundamentals of Forensic Anthropology.

[3] Galeta P., Brůžek J., Obertová Z., Cattaneo C., Stewart A. (2020). Sex estimation using continuous variables: Problems and principles of sex classification in the zone of uncertainty. Statistics and Probability in Forensic Anthropology.

[4] Cabo L.L., Brewster C.P., Luengo Azpiazu J. (2012). Sexual dimorphism: Interpreting sex markers. Companion Forensic Anthropol..

[5] Brůžek J., Santos F., Dutailly B., Murail P., Cunha E. (2017). Validation and reliability of the sex estimation of the human os coxae using freely available DSP2 software for bioarchaeology and forensic anthropology. Am. J. Phys. Anthropol..

[6] D’Oliveira Coelho J., Curate F. (2019). CADOES: An interactive machine-learning approach for sex estimation with the pelvis. Forensic Sci. Int..

[7] Murail P., Bruzek J., Braga J. (1999). A new approach to sexual diagnosis in past populations. Practical adjustments from Van Vark’s procedure. Int. J. Osteoarchaeol..

[8] Avent P.R., Hughes C.E., Garvin H.M. (2022). Applying posterior probability informed thresholds to traditional cranial trait sex estimation methods. J. Forensic Sci..

[9] Santos F., Guyomarc’h P., Bruzek J. (2014). Statistical sex determination from craniometrics: Comparison of linear discriminant analysis, logistic regression, and support vector machines. Forensic Sci. Int..

[10] Milner G.R., Boldsen J.L. (2012). Humeral and femoral head diameters in recent white American skeletons. J. Forensic Sci..

[11] Jerković I., Bašić Ž., Anđelinović Š., Kružić I. (2020). Adjusting posterior probabilities to meet predefined accuracy criteria: A proposal for a novel approach to osteometric sex estimation. Forensic Sci. Int..

[12] Hussein M.H.A., Abulnoor B.A.E.-S. (2019). Sex estimation of femur using simulated metapopulation database: A preliminary investigation. Forensic Sci. Int. Rep..

[13] Attia M.H., Attia M.H., Farghaly Y.T., Abulnoor B.A.E.-S., Curate F. (2022). Performance of the supervised learning algorithms in sex estimation of the proximal femur: A comparative study in contemporary Egyptian and Turkish samples. Sci. Justice.

[14] Curate F., Umbelino C., Perinha A., Nogueira C., Silva A.M., Cunha E. (2017). Sex determination from the femur in Portuguese populations with classical and machine-learning classifiers. J. Forensic Leg. Med..

[15] Attia M.H., Aboulnoor B.A.E. (2020). Tailored logistic regression models for sex estimation of unknown individuals using the published population data of the humeral epiphyses. Leg. Med..

[16] Bartholdy B.P., Sandoval E., Hoogland M.L.P., Schrader S.A. (2020). Getting Rid of Dichotomous Sex Estimations: Why Logistic Regression Should be Preferred Over Discriminant Function Analysis. J. Forensic Sci..

[17] Papaioannou V.A., Kranioti E.F., Joveneaux P., Nathena D., Michalodimitrakis M. (2012). Sexual dimorphism of the scapula and the clavicle in a contemporary Greek population: Applications in forensic identification. Forensic Sci. Int..

[18] Hora M., Sládek V. (2018). Population specificity of sex estimation from vertebrae. Forensic Sci. Int..

[19] Navega D., Vicente R., Vieira D.N., Ross A.H., Cunha E. (2015). Sex estimation from the tarsal bones in a Portuguese sample: A machine learning approach. Int. J. Leg. Med..

[20] Konigsberg L.W., Frankenberg S.R. (2019). Multivariate ordinal probit analysis in the skeletal assessment of sex. Am. J. Phys. Anthropol..

[21] Konigsberg L.W., Algee-Hewitt B.F., Steadman D.W. (2009). Estimation and evidence in forensic anthropology: Sex and race. Am. J. Phys. Anthropol..

[22] Jantz R.L., Ousley S.D., Klales A.R. (2020). Sexual dimorphism variation in Fordisc samples. Sex Estimation of the Human Skeleton.

[23] Buikstra J.E. (1994). Standards for Data Collection from Human Skeletal Remains: Proceedings of a Seminar at the Field Museum of Natural History.

[24] Moore-Jansen P.H., Jantz R.L. (1994). Data Collection Procedures for Forensic Skeletal Material.

[25] Jerković I., Kolić A., Kružić I., Anđelinović Š., Bašić Ž. (2021). Adjusted binary classification (ABC) model in forensic science: An example on sex classification from handprint dimensions. Forensic Sci. Int..

[26] Gulhan O. (2017). Skeletal Sexing Standards of Human Remains in Turkey. Ph.D. Thesis.

[27] Gregory J.S., Aspden R.M. (2008). Femoral geometry as a risk factor for osteoporotic hip fracture in men and women. Med. Eng. Phys..

[28] Terzidis I., Totlis T., Papathanasiou E., Sideridis A., Vlasis K., Natsis K. (2012). Gender and Side-to-Side Differences of Femoral Condyles Morphology: Osteometric Data from 360 Caucasian Dried Femori. Anat. Res. Int..

[29] Ul-Haq Z., Madura J.D. (2015). Frontiers in Computational Chemistry: Volume 2: Computer Applications for Drug Design and Biomolecular Systems.

[30] Ferrer A.J.A., Wang L. Comparing the classification accuracy among nonparametric, parametric discriminant analysis and logistic regression methods. Proceedings of the 1 Annual Meeting of the American Educational Research Association.

[31] Kuhn M., Wing J., Weston S., Williams A., Keefer C., Engelhardt A., Cooper T., Mayer Z., Kenkel B., Team R.C. (2020). Package ‘caret’. R J..

[32] Wickham H., Francois R., Henry L., Müller K. (2015). dplyr: A Grammar of Data Manipulation. R package Version 0.4.3.

[33] Pedersen T. (2017). Patchwork: The Composer of ggplots. R Package Version 0.0.1.

[34] Kassambara A. (2020). rstatix: Pipe-Friendly Framework for Basic Statistical Tests. R package Version 0.6.0.

[35] Wickham H., Averick M., Bryan J., Chang W., McGowan L.D.A., François R., Grolemund G., Hayes A., Henry L., Hester J. (2019). Welcome to the Tidyverse. J. Open Source Softw..

[36] Kassambara A. (2018). ggpubr:“ggplot2” Based Publication Ready Plots (Version 0.1.7). https://CRAN.R-project.org/package=ggpubr.

[37] Leisch F. (2009). mlbench: Machine Learning Benchmark Problems. R Package Version.

[38] Pastore M. (2018). Overlapping: A R package for estimating overlapping in empirical distributions. J. Open Source Softw..

[39] Sarkar D., Sarkar M.D. (2007). The Lattice Package. Trellis Graphics for R. https://cran.r-project.org/web/packages/lattice/lattice.pdf.

[40] Smith B. (2020). MachineShop: Machine Learning Models and Tools. R Package Version. https://cran.r-project.org/web/packages/MachineShop/MachineShop.pdf.

[41] Brownlee J. (2019). Feature Selection with the Caret R Package. https://machinelearningmastery.com/feature-selection-with-the-caret-r-package/.

[42] Nikita E., Nikitas P. (2020). On the use of machine learning algorithms in forensic anthropology. Leg. Med..

[43] Toneva D., Nikolova S., Agre G., Zlatareva D., Hadjidekov V., Lazarov N. (2021). Machine learning approaches for sex estimation using cranial measurements. Int. J. Leg. Med..

[44] Tutz G., Binder H. (2006). Generalized additive modeling with implicit variable selection by likelihood-based boosting. Biometrics.

[45] Williams G. (2006). Data Mining Desktop Survival Guide. Usage2. html.

[46] Akter T., Satu M.S., Khan M.I., Ali M.H., Uddin S., Lio P., Quinn J.M., Moni M.A. (2019). Machine learning-based models for early stage detection of autism spectrum disorders. IEEE Access.

[47] Lopes M. Is LDA a Dimensionality Reduction Technique or a Classifier Algorithm. https://towardsdatascience.com/is-lda-a-dimensionality-reductiontechnique-or-a-classifier-algorithm-eeed4de9953a.

[48] Ripley B., Venables B., Bates D.M., Hornik K., Gebhardt A., Firth D., Ripley M.B. (2013). Package ‘mass’. CRAN R.

[49] Iworiso J. (2020). On the Predictability of US Stock Market Using Machine Learning and Deep Learning Techniques. Ph.D. Thesis.

[50] Hind J., Hussain A., Al-Jumeily D., Montañez C.A.C., Chalmers C., Lisboa P. Robust interpretation of genomic data in chronic obstructive pulmonary disease (COPD). Proceedings of the 2018 11th International Conference on Developments in eSystems Engineering (DeSE).

[51] Hofner B., Mayr A., Robinzonov N., Schmid M. (2014). Model-based boosting in R: A hands-on tutorial using the R package mboost. Comput. Stat..

[52] Olson D.L., Wu D. (2017). Predictive Data Mining Models.

[53] Lin H.-T., Lin C.-J., Weng R.C. (2007). A note on Platt’s probabilistic outputs for support vector machines. Mach. Learn..

[54] Karatzoglou A., Smola A., Hornik K., Zeileis A. (2004). kernlab—An S4 package for kernel methods in R. J. Stat. Softw..

[55] Bolger F., Wright G. (1992). Reliability and validity in expert judgment. Expertise and Decision Support.

[56] Sordo M., Zeng Q., Oliveira J.L., Maojo V., Martin-Sanchez F., Pereira A.S. (2005). On sample size and classification accuracy: A performance comparison. Proceedings of the 6th International Symposium on Biological and Medical Data Analysis ISBMDA 2005.

[57] Zhang Y., Ling C. (2018). A strategy to apply machine learning to small datasets in materials science. NPJ Comput. Mater..

[58] Lei P.-W., Koehly L.M. (2003). Linear discriminant analysis versus logistic regression: A comparison of classification errors in the two-group case. J. Exp. Educ..

[59] Pohar M., Blas M., Turk S. (2004). Comparison of logistic regression and linear discriminant analysis: A simulation study. Metodoloski Zv..

[60] Mansournia M.A., Geroldinger A., Greenland S., Heinze G. (2018). Separation in Logistic Regression: Causes, Consequences, and Control. Am. J. Epidemiol..

[61] Stephan C.N., Norris R.M., Henneberg M. (2005). Does sexual dimorphism in facial soft tissue depths justify sex distinction in craniofacial identification?. J. Forensic Sci..

[62] Raza H., Cecotti H., Li Y., Prasad G. (2016). Adaptive learning with covariate shift-detection for motor imagery-based brain–computer interface. Soft Comput..

[63] Parikh R., Mathai A., Parikh S., Chandra Sekhar G., Thomas R. (2008). Understanding and using sensitivity, specificity and predictive values. Indian J. Ophthalmol..

[64] Ortega R.F., Irurita J., Campo E.J.E., Mesejo P. (2021). Analysis of the performance of machine learning and deep learning methods for sex estimation of infant individuals from the analysis of 2D images of the ilium. Int. J. Leg. Med..

[65] Cao Y., Ma Y., Yang X., Xiong J., Wang Y., Zhang J., Qin Z., Chen Y., Vieira D.N., Chen F. (2022). Use of deep learning in forensic sex estimation of virtual pelvic models from the Han population. Forensic Sci. Res..

