# Supplementary material for: Efficiency of the Adjusted Binary Classification (ABC) Approach in Osteometric Sex Estimation: A Comparative Study of Different Linear Machine Learning Algorithms and Training Sample Sizes
Source: Biology (Basel). 2022 Jun 15;11(6):917. doi: 10.3390/biology11060917 (PMC9220275; doi:10.3390/biology11060917)
Supplement: Supplementary file 1 [file biology-11-00917-s001.zip › ABC supplemantary figures.pdf]

Supplementary Figures

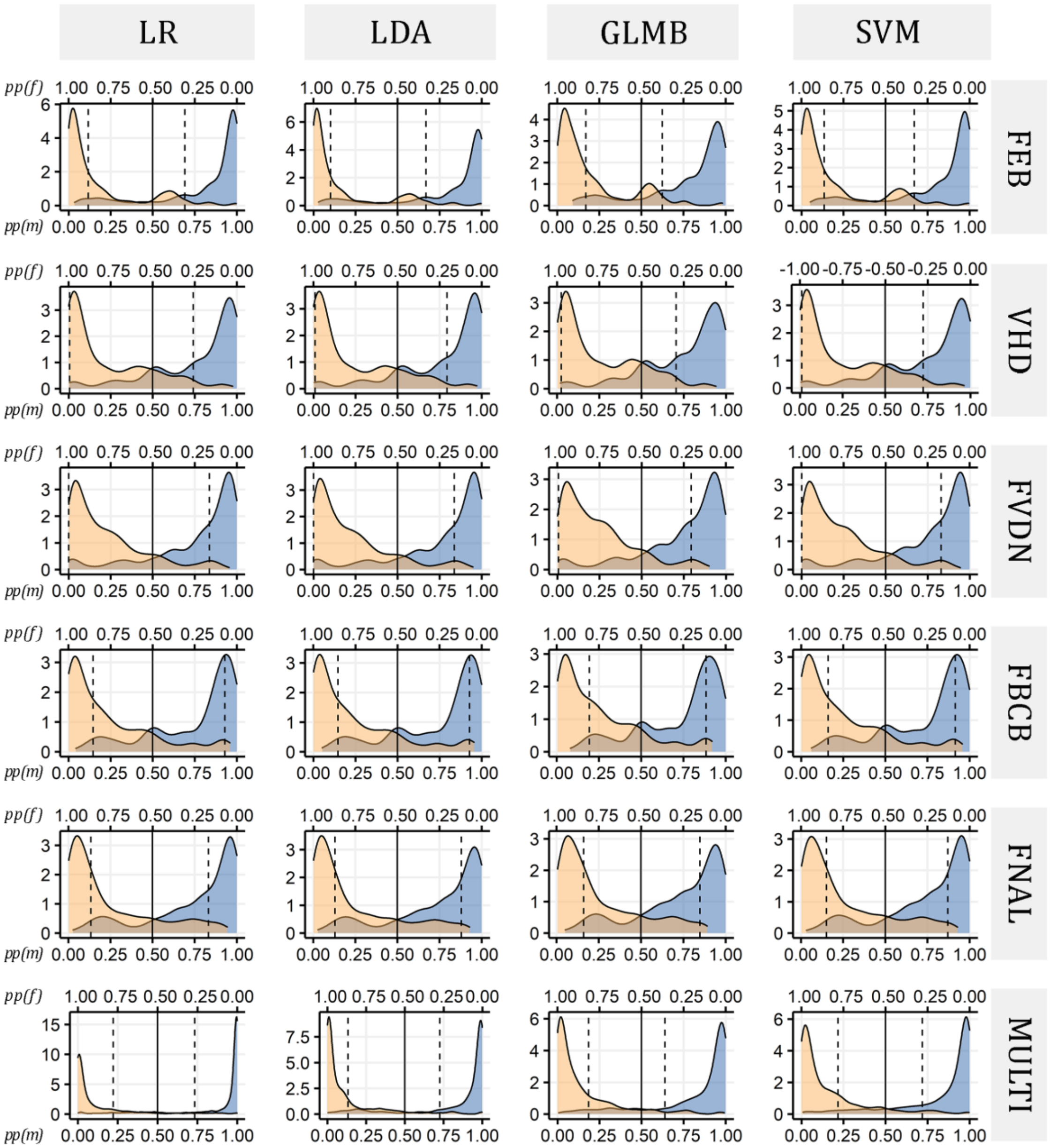

Supplementary Figure S1. Comparison between the estimated posterior classification probability densities of the two sex groups in the training set. The overlap is represented by the shaded area. The continuous black line identifies the default pp=0.5 used for classification whereas the interrupted lines represent the limits of the pp thresholds calculated using the ABC method for each classifier and model.

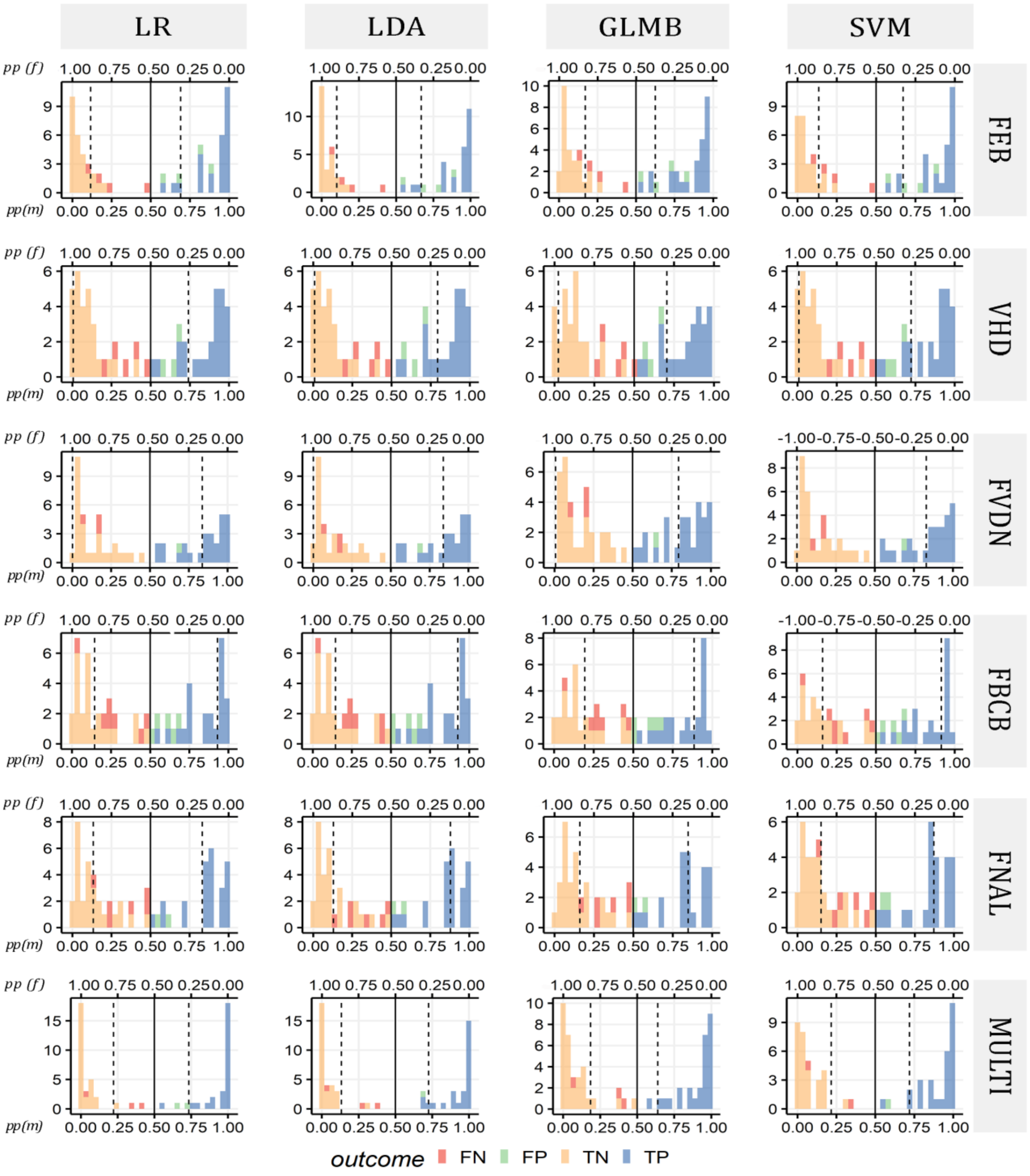

Supplementary Figure S2. Plot of the posterior probabilities (pp) values as a histogram in terms of individual frequencies. For every pp value, the number of TP, FP, TN, and FN cases is exhibited on the x-axis. Each group (true positives=males/true negatives=females) was displayed in different colors (blue and yellow, respectively). The individuals present within the posterior probabilities' cutoff limits (interrupted lines) were not classifiable. Above these pp thresholds, true positive and negative samples were displayed. However, false negative (red) samples are present within the female region and false positive (green) samples exist within the male region and their corresponding pp values are shown on their relevant scale at the top and bottom of each panel
